# Fish Segmentation in Sonar Images by Mask R-CNN on Feature Maps of Conditional Random Fields †

**DOI:** 10.3390/s21227625

**Published:** 2021-11-17

**Authors:** Chin-Chun Chang, Yen-Po Wang, Shyi-Chyi Cheng

**Affiliations:** Department of Computer Science and Engineering, National Taiwan Ocean University, 2, Pei-Ning Rd., Keelung 202301, Taiwan; cvml@mail.ntou.edu.tw (C.-C.C.); cvml@email.ntou.edu.tw (Y.-P.W.)

**Keywords:** fish segmentation, sonar images, conditional random fields, mask R-CNN

## Abstract

Imaging sonar systems are widely used for monitoring fish behavior in turbid or low ambient light waters. For analyzing fish behavior in sonar images, fish segmentation is often required. In this paper, Mask R-CNN is adopted for segmenting fish in sonar images. Sonar images acquired from different shallow waters can be quite different in the contrast between fish and the background. That difference can make Mask R-CNN trained on examples collected from one fish farm ineffective to fish segmentation for the other fish farms. In this paper, a preprocessing convolutional neural network (PreCNN) is proposed to provide “standardized” feature maps for Mask R-CNN and to ease applying Mask R-CNN trained for one fish farm to the others. PreCNN aims at decoupling learning of fish instances from learning of fish-cultured environments. PreCNN is a semantic segmentation network and integrated with conditional random fields. PreCNN can utilize successive sonar images and can be trained by semi-supervised learning to make use of unlabeled information. Experimental results have shown that Mask R-CNN on the output of PreCNN is more accurate than Mask R-CNN directly on sonar images. Applying Mask R-CNN plus PreCNN trained for one fish farm to new fish farms is also more effective.

## 1. Introduction

In modern aquaculture, fish states are constantly monitored to ensure the health of the cultured fish. Because computer vision can provide noninvasive means of monitoring, computer vision-based systems have been developed for a variety of applications in aquaculture [1,2].

**Figure 1:**
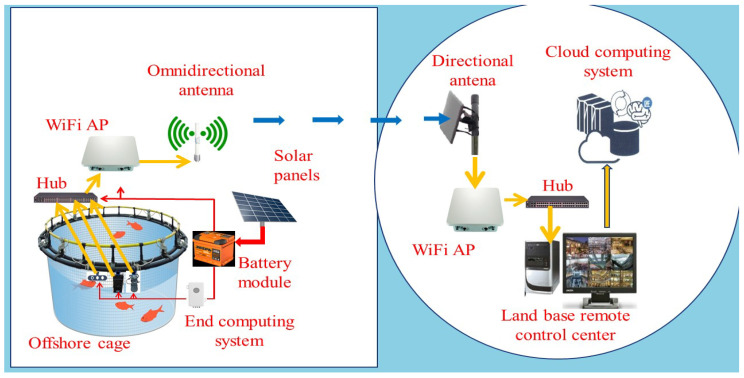
The proposed AIoT based smart cage system using multi-mode sensors include a stereo camera, a sonar imaging camera, and a set of water quality detectors. The time series data of each sensor device are first processed by the associate end computing system and sent to the cloud platform which offers all the AI micro-service computing for precise aquaculture.

Figure 1 shows our AIoT based smart aquaculture system in which both the RGB camera and the sonar imaging device are used to capture the underwater images of fish inside an offshore cage. Our sonar imaging device helps to monitor the health condition of the fish when the lighting condition is poor, which often limits the usage of RGB cameras to capture clear images for fish monitoring. To achieve the goal of smart aquaculture, fish counting and fish body length estimation based on underwater images are the two essential functionalities. Both of them are important to estimate the growth curve of fish and the feeding amount of an aquaculture cage to achieve the goal of precise aquaculture. The Mask R-CNN deep learning model offers the fish detection and fish segmentation simultaneously based on the captured underwater sonar images. The results could be further used to count fish and estimate the body length of the fish in a non-intrusive manner. Non-intrusive methods can reduce the manual handling of the fish, thus, can prevent stress and disturbance among the fish school. It is in this sense that we integrated a non-contact and visual method to estimate the fish biological information specifically on its body length and biomass that can avoid fish injury and illness caused by fish catching to estimate the fish biological information. Although semantic object segmentation based on a CNN deep learning model and underwater images is not a new concept, to the best of our knowledge, the topic of this paper and the sensing technology for analyzing fish in sonar images are rarely studied.

**Figure 2:**
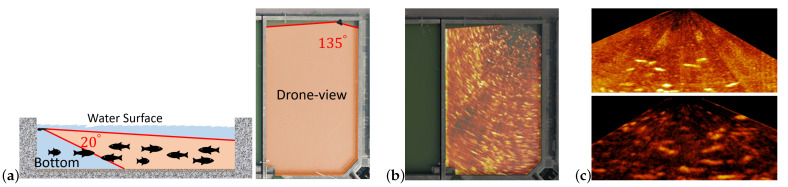
Illustrations of sonar images acquired from fish farms by the Garmin CHIRP imaging sonar system, where (**a**) depicts the underwater area covered by the imaging sonar system; (**b**) is a drone-view of a fish pond overlapped with the rectified sonar image; (**c**) shows two sonar images acquired from different land-based fish farms.

The visual quality of underwater images can be poor because light can be heavily attenuated and scattered in water. Underwater image enhancement is usually required for analysis of the image of underwater objects [3,4]. On the other hand, imaging sonar systems often apply to fish monitoring in turbid or low ambient light waters [5,6,7,8,9]. Figure 2a depicts the underwater area covered by the imaging sonar system adopted in this paper. Figure 2b shows a drone view of a land-based fish farm overlayed with the rectified underwater sonar image. In sonar images, fish are often overlapping and the pixel value is proportional to the intensity of the received reflection of the sonar signal. In land-based fish farms, the sonar signal can be reflected from the fish, the facility, and the bottom and wall of the fish farm. Different materials can have different reflectivity for underwater sound. For example, the reflectivity of the fish is related to the species of the fish [9]. The reflectivity of sand is higher than that of mud [10]. The distance from the sonar system to an object also affects the intensity of the echo from this object. Even if the gain setting on the imaging sonar system is properly tuned, sonar images acquired from fish farms of different size, depth, and structural materials can be quite different in the contrast between fish and the background.

Fish segmentation is often required for computer vision-based monitoring of fish growth and behavior. Deep convolutional neural networks (CNNs) for instance segmentation, such as SGN [11], FCIS [12], Mask R-CNN [13], TensorMask [14], have shown excellent performances. Those CNNs can usually be transferred by fine-tuning to segment other target instances. In this paper, Mask R-CNN is adopted for fish segmentation in sonar images.

As Figure 2c shows, sonar images acquired from different land-based fish farms can be quite different in the echo from the fish and the bottom. That difference can make Mask R-CNN, which is fine-tuned based on training examples of one fish farm, ineffective to fish segmentation for the other fish farms. A method for fine-tuning and generalizing Mask R-CNN for fish segmentation in sonar images is worthy of investigation.

**Figure 3:**
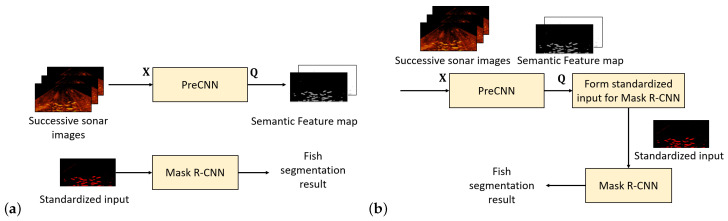
The flow of Mask R-CNN+PreCNN: (**a**) the flow of training PreCNN and fine-tuning Mask R-CNN; (**b**) the flow of fish segmentation.

Figure 3 presents the proposed approach. A preprocessing CNN (PreCNN) for Mask R-CNN is proposed so that Mask R-CNN+PreCNN learned for one fish-cultured environment can effectively apply to new environments. PreCNN is a semantic segmentation network and integrated with conditional random fields (CRFs). PreCNN and Mask R-CNN can be separately trained and fine-tuned. As Figure 3a shows, the input of PreCNN comprises several successive frames of a sonar video. PreCNN outputs a two-channel semantic feature map, which represents the estimated posterior probabilities of each pixel belonging to the background and the fish. The semantic feature map is used to form a standardized three-channel input for Mask R-CNN, which comprises the fish-channel of the semantic feature map and two channels of zeros. Mask R-CNN is fine-tuned on the standardized input for fish segmentation. Figure 3b shows the flow of fish segmentation, where PreCNN, the module for forming the standardized input for Mask R-CNN, and Mask R-CNN sequentially apply. By considering the neural architectures for Mask R-CNN and PreCNN as a whole, the output of PreCNN is a semantic intermediate representation of successive sonar images. The proposed approach explicitly learns the semantic feature mapping, which has good potential for crossing different fish-cultured environments.

Mask R-CNN+PreCNN has the advantages:

- Decoupling learning of fish instances from learning of fish-cultured environments: PreCNN learns a mapping from sonar images to a semantic feature map. Mask R-CNN is fine-tuned on the semantic feature map. Thus, learning of fish instances and learning of fish-cultured environments can be separated.
- Utilizing temporal information in successive sonar-image frames: In noisy sonar images, fish identification is usually more accurate by multiple frames than by a single frame;
- Semi-supervised learning: To reduce annotation costs, ambiguous pixels and pixels similar to annotated background pixels are not required to annotate. Images with partial or no pixel-level annotations can be used to train PreCNN in a semi-supervised learning manner.

Experimental results have shown that PreCNN can improve the accuracy of Mask R-CNN for fish segmentation, especially across different fish-cultured environments.

This paper is organized as follows. Related works are presented in Section 2. PreCNN is presented in Section 3. An extension of PreCNN, which can provide useful information for Mask R-CNN to segment overlapping fish, is also presented there. Experimental results are discussed in Section 4. Concluding remarks are drawn in Section 5.

## 2. Related Work

To monitor the states of fish in underwater areas with low optical visibility, imaging sonar systems are often without alternative. Applications of imaging sonar systems in aquaculture are broad, such as fish counting [5,15,16,17,18,19], recording fish schools [9,20], fish tracking [21], fish detection [8,22], and monitoring of fish behavior [6,7] and feeding [23,24]. Image processing algorithms, such as adaptive thresholding and background subtraction, often apply in those applications for fish segmentation. However, those algorithms are often sensitive to noise and sonar artifacts [25].

Object detection and image segmentation algorithms have been developed to detect objects in sonar images. In [26,27,28], unsupervised learning algorithms and likelihood ratio tests are proposed to separate the highlight and shadow regions of unknown objects from the background seabed. In [29], CNNs are found suitable for detecting objects of known shapes on the seabed. In [18,19], CNNs are also shown to be effective in fish counting in sonar images.

Mask R-CNN is widely used for instance segmentation in optical images, such as fish detection [30] and ship detection [31]. Mask R-CNN belongs to the currently dominant paradigm for instance segmentation—the detect-then-segment methodology [14]. According to the taxonomy of the instance segmentation networks [14,32], there are backbone networks extracting image features for object detection and segmentation. In sonar images, those backbone networks can couple fish instances with fish-cultured environments. In this paper, to decouple learning of fish instances from learning of fish-cultured environments in sonar images, PreCNN is developed to provide for Mask R-CNN the semantic information in sonar images.

Many CNNs for semantic segmentation [13,33,34,35,36] for optical or medical images have come out. A comprehensive review of semantic image segmentation by deep learning models can be found in [37]. In FCN [34], pre-trained CNNs for image classification are casted into a fully convolutional form for pixel-level classification. Full resolution feature maps are recovered by a upsampling network, which can combine information from shallow layers and deep layers by skip connections. Transposed convolution is widely used in the upsampling network. A drawback of transposed convolution is the checkerboard problem. This drawback can be coped with pixel-transposed convolution [38]. In DeepLab [35], it turns out that CNNs abstracting spatial information by successive max-pooling and downsampling can lose the spatial accuracy. Dilated (Atrous) convolution is thus introduced to enlarge the receptive field without a loss of feature map resolution. DeepLab also applies a fully connected CRF to improve the spatial consistency of the segmentation result.

In [39], it turns out that CNNs and dense CRFs can be trained in an end-to-end manner by formulating CRFs as recurrent neural networks (RNNs). The pairwise potentials in [39] are limited to weighted Gaussians on predefined image features. In [40], CNNs are combined with a Gaussian CRF network, where all parameters are trainable. Freeform pairwise potentials with all parameters trainable have come out, such as [36,41].

The attention mechanism has been applied to semantic segmentation. In [42], an attention model is proposed to learn to softly weights the multi-scale features when predicting the semantic label of the pixel. In [43], a position attention module and a channel attention module are appended on the top of a dilated FCN to learn the semantic interdependencies in spatial and channel dimensions, respectively.

Due to the high cost of pixel-level annotations, weakly- and semi-supervised learning algorithms of semantic segmentation, such as [44,45,46], have been proposed. The training set for those algorithms can comprise training examples with strong and weak pixel-level annotations and image-level annotations. On the other hand, few-shot semantic segmentation can segment test images given only a few annotated support images [47,48]. If there exist a lot of unlabeled and related examples, self-training can improve the semantic segmentation model [49]. However, those two approaches are out of the scope of the application considered in this paper.

In summary, sonar images can be noisy in shallow waters. CRFs can be integrated into PreCNN to get spatial consistent label maps. Since it is not easy to empirically set parameters for CRFs on sonar images, freeform pairwise potentials for CRFs with all parameters trainable are required. Besides, to get rid of laboriously annotating every pixel of a sonar image, end-to-end training for PreCNN in a semi-supervised learning manner is also preferable.

## 3. Materials and Methods

### 3.1. Problem Formulation

Let L denote a label set L = {1,…,ℓ}. In an annotation image, a labelled pixel can be either a pixel outside a fish or a pixel in a fish. To give fish motion information in annotation images, the label of a pixel in a fish can also be related to the motion direction of the fish. That extension will be presented in Section 3.5.

Let X denote a multiple-channel image which is formed by stacking a sequence of one-channel sonar images. In this paper, X comprises three successive sonar images. Let Y denote the label map assigned to X and $y_{i}$ be the label of pixel *i*. The probability of Y given X in a CRF can be modeled by the Gibbs distribution as [36,39]

$$P\left(Y|X\right)\;=\;\frac{1}{Z(X)}\exp\left(-E\left(Y|X\right)\right),$$

where Z(X) is the partition function and E(Y|X) is the Gibbs energy defined as

(1) $$E\left(Y|X\right)\;=\;\sum_{i}\psi_{u}\left(y_{i}|X\right)\;+\;\sum_{i}\sum_{j\in N_{i}}\psi_{p}\left(y_{i},y_{j}|X\right).$$

In Equation (1), $N_{i}$ denotes a set of neighboring pixels of pixel *i*, $\psi_{u}\left(y_{i}|X\right)$ denotes the unary potential, and $\psi_{p}\left(y_{i},y_{j}|X\right)$ is the pairwise potential. The unary potential $\psi_{u}\left(y_{i}|X\right)$ is the cost of assigning label $y_{i}$ to pixel *i* and can be defined on the output of a deep CNN $\phi_{u}\left(y_{i}|X\right)$ as

$$\psi_{u}\left(y_{i}|X\right)=-\log\left(\phi_{u}\left(y_{i}|X\right)\right).$$

The deep CNN $\phi_{u}\left(y_{i}|X\right)$ will be defined later. The pairwise potential $\psi_{p}\left(y_{i},y_{j}|X\right)$ is the cost of assigning labels $y_{i}$ and $y_{j}$, respectively, to pixels *i* and *j*. The potential $\psi_{p}\left(y_{i},y_{j}|X\right)$ can be defined as [36]

$$\psi_{p}\left(y_{i},y_{j}|X\right)\;=\;c\left(y_{i},y_{j}\right)f\left(f_{i},f_{j},d_{ij}\right),$$

where c(u,v) is the compatibility from label *v* to label *u* and $f(X_{i},X_{j},d_{ij})$ is the similarity between pixels *i* and *j* in terms of image features $f_{i}$ and $f_{j}$ and the spatial distance $d_{i,j}$ between pixels *i* and *j*. The deep feature $f_{i}$ is extracted by $\phi_{u}\left(y_{i}|X\right)$. In the proposed approach, c(u,v), $f(f_{i},f_{j},d_{ij})$, and $\phi_{u}\left(y_{i}|X\right)$ are all trainable.

### 3.2. The Mean-Field Approximation to P(Y|X)

For efficient inference of the CRF, the mean-field approximation Q(Y|X) to the distribution P(Y|X) often applies [36,39,40], where Q(Y|X) is of the fully factorized form

$$Q\left(Y|X\right)\;=\;\prod_{i}Q_{i}\left(y_{i}|X\right)$$

and minimizes the Kullback-Leibler (KL) divergence $D_{KL}\left(Q||P\right)$. The distribution $Q_{i}\left(y_{i}|X\right)$ can be obtained by

(2) $$Q_{i}\left(y_{i}|X\right)\;=\;\frac{1}{Z_{i}}\exp\left(-\left(\psi_{u}\left(y_{i}|X\right)\;+\;\phi_{p}\left(y_{i}|X\right)\right)\right)$$

where $Z_{i}$ is the local normalization constant and

$$\phi_{p}\left(y_{i}\;=\;u|X\right)\;=\;\sum_{v\in L}c\left(u,v\right)\sum_{j\in N_{i}}f\left(f_{i},f_{j},d_{ij}\right)Q_{j}\left(y_{j}=v|X\right).$$

Equation (2) can be turned into a fixed-point form as

(3) $$Q_{i}^{\left(t\right)}\left(y_{i}|X\right)\;=\;\frac{1}{Z_{i}^{\left(t\right)}}\exp\left(-\left(\psi_{u}\left(y_{i}|X\right)\;+\;\phi_{p}^{\left(t-1\right)}\left(y_{i}|X\right)\right)\right)$$

where

(4) $$\phi_{p}^{\left(t-1\right)}\left(y_{i}\;=\;u|X\right)\;=\;\sum_{v\in L}c\left(u,v\right)\sum_{j\in N_{i}}f\left(f_{i},f_{j},d_{ij}\right)Q_{j}^{\left(t-1\right)}\left(y_{j}\;=\;v|X\right)$$

with

$$Q_{i}^{\left(0\right)}\left(y_{i}|X\right)\;=\;\frac{1}{Z_{i}^{\left(0\right)}}\exp\left(-\psi_{u}\left(y_{i}|X\right)\right).$$

The distribution function $Q_{i}\left(y_{i}|X\right)$ for all pixels can be updated in parallel.

### 3.3. Semi-Supervised Learning

The distribution function Q(Y|X) can be learned in a manner of semi-supervised learning. For clarity, Θ denotes all parameters to learn for Q(Y|X) and Q(Y|X) is added by a subscript notation as $Q_{\Theta }\left(Y|X\right)$. Let $H_{s}$ be the annotation image for training example $X_{s}$, and $h_{s;i;u}$ be the variable for indicating if the label of pixel *i* of $X_{s}$ is *u*. If pixel *i* of $X_{s}$ is manually annotated, $h_{s;i;u}\in \left\{0,1\right\}$ are all constant; otherwise, $h_{s;i;u}\in \left[0,1\right]$ are all latent variables. Additionally, we have $\sum_{u\in L}h_{s;i;u}\;=\;1$. The complete data likelihood function can be defined as

$$L_{c}\left(\Theta \right)\;=\;\prod_{s}\prod_{i}\prod_{u\in L}Q_{i;\Theta }^{h_{s;i;u}}\left(y_{i}\;=\;u|X_{s}\right),$$

and the complete data log-likelihood function is

$$l_{c}\left(\Theta \right)\;=\;\sum_{s}\sum_{i}\sum_{u\in L}h_{s;i;u}\log\left(Q_{i;\Theta }\left(y_{i}\;=\;u|X_{s}\right)\right).$$

According to the EM algorithm, Θ can be estimated by maximizing the expected complete data log-likelihood function:

$$\Theta^{\left(t\right)}\;=\;\underset{\Theta }{\mathrm{error}}\pi \left(\Theta |\Theta^{\left(t-1\right)}\right)$$

where

$$\begin{array}{cc} \pi \left(\Theta |\Theta^{\left(t-1\right)}\right)\;=\;\sum_{s} & \left(\sum_{i\notin A_{s}}\sum_{u\in L}E_{Q_{i;\Theta^{\left(t-1\right)}}}\left[h_{s;i;u}\right]\log\left(Q_{i;\Theta }\left(y_{i}\;=\;u|X_{s}\right)\right)\;+\;\right.\\ & \left.C\times \sum_{i\in A_{s}}\sum_{u\in L}h_{s;i;u}\log\left(Q_{i;\Theta }\left(y_{i}\;=\;u|X_{s}\right)\right)\right) \end{array}$$

with $A_{s}$ the set of labeled pixels of $X_{s}$ and

$$E_{Q_{i;\Theta^{\left(t-1\right)}}}\left[h_{s;i;u}\right]\;=\;Q_{i;\Theta^{\left(t-1\right)}}\left(y_{i}\;=\;u|X_{s}\right).$$

In $\pi (\Theta |\Theta^{\left(t-1\right)})$, the labelled pixel is weighted by a factor *C*, which is set to 1 in this paper. A mini-batch gradient-descent algorithm with the loss function $-\pi (\Theta |\Theta^{\left(t-1\right)})$ can be adopted to learn Θ.

### 3.4. The Neural Network Architecture

**Figure 4:**
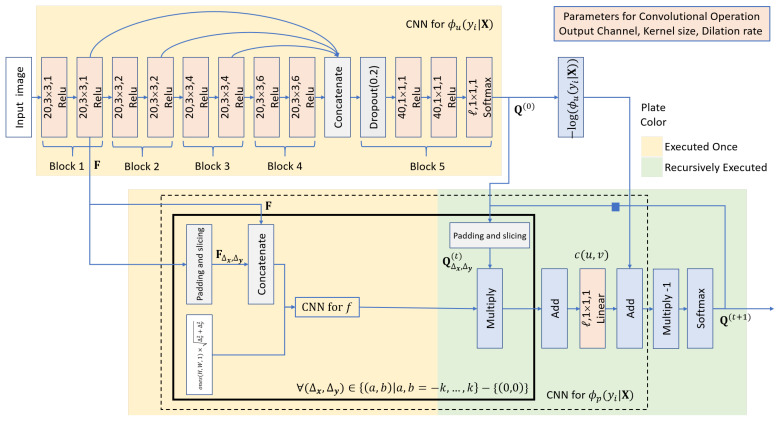
The neural architecture of $Q(y_{i}|X)$, where $F_{\Delta_{x},\Delta_{y}}$ denotes the feature map, which is of the same size of F and covers starting from the $k\;+\;\Delta_{y}$th row and the $k\;+\;\Delta_{x}$th column of F with *k* padded zeros at the top, bottom, left, and right side; in addition, “$\forall (\Delta_{x},\Delta_{y})$” at the corner of the rectangle with solid lines indicates that the network inside is performed for each $\left(\Delta_{x},\Delta_{y}\right)$.

As Figure 4 shows, the distribution function Q(Y|X) can be implemented by a deep neural network, which comprises four parts for the four functions:

- $\phi_{u}\left(y_{i}|X\right)$: a deep CNN;
- $\phi_{p}\left(y_{i}|X\right)$: an RNN;
- $f(f_{i},f_{j},d_{ij})$: a CNN comprising a sequence of 1×1 convolutional operations;
- c(u,v): a 1×1 convolutional operation.

The distribution function $\phi_{u}\left(y_{i}|X\right)$ is implemented by a deep CNN. This deep CNN consists of five blocks and its network architecture is similar to the P-Net [36]. The first four blocks comprise 3 × 3 dilated convolutional layers with dilation rates 1, 2, 4, and 6, respectively. Dilated convolutional operations are adopted to enlarge receptive fields without a loss of feature map resolution. The outputs of the last layers of the first four blocks are concatenated together to be the input for the fifth block. The fifth block comprises one dropout layer and three 1 × 1 convolutional layers.

The mean-field CRF inference, Equation (3), can be implemented as an RNN [39]. By padding, slicing, and concatenating the feature map from the first block of the CNN for $\phi_{u}\left(y_{i}|X\right)$, the two functions *f* and *c* in Equation (4) can be implemented by 1 × 1 convolutional operations. The output of $f(f_{i},f_{j},d_{ij})$ is a product of two sigmoid functions for indicating if pixels *i* and *j* are nearby pixels and similar in image features. The function c(u,v) can be implemented by a 1 × 1 convolutional operation with nonnegative weights, zero bias terms, and linear activation functions. Due to the memory space, $N_{i}$ only includes the pixel in the 3 × 3 neighborhood of pixel *i* in this paper.

### 3.5. Segmentation of Overlapping Fish with Mask R-CNN

In addition to fish shapes [50], the fish motion direction is also a cue for segmenting overlapping fish. PreCNN${}^{d}$, which is an extension of PreCNN, provides motion information for Mask R-CNN for segmenting overlapping fish. In an annotation image for training PreCNN${}^{d}$, a fish pixel can be annotated by the category of the motion direction of the fish. In this paper, the fish motion direction is categorized into four directions: the north-west, north-east, south-west, and south-east directions. Thus, the output Q of PreCNN${}^{d}$ is a 5-channel feature map, where one channel is for the background and the others are for the four motion direction categories.

To make Mask R-CNN utilize the fish motion direction, the output of PreCNN${}^{d}$ is combined channel-wisely to provide multiple standardized inputs for Mask R-CNN. Fifteen combinations of the four motion-direction categories (the empty combination is excluded) can be considered. Thus, fifteen standardized 3-channel inputs for Mask R-CNN are created. A 3-channel standardized input comprises two channels of zeros and a channel which is the channel-wise sum of Q across the channels corresponding to the considered motion-direction categories. Each input presents a possible interpretation of fish shapes according to some motion directions. At last, apply the non-maximum-suppression technique to all detected fish masks to get the final results. Accordingly, the fish size can be estimated based on non-overlapping fish. In Mask R-CNN+PreCNN${}^{d}$, Mask R-CNN can be trained on the standardized input with the four fish-motion categories all considered.

## 4. Results

### 4.1. Test Environments

**Figure 5:**
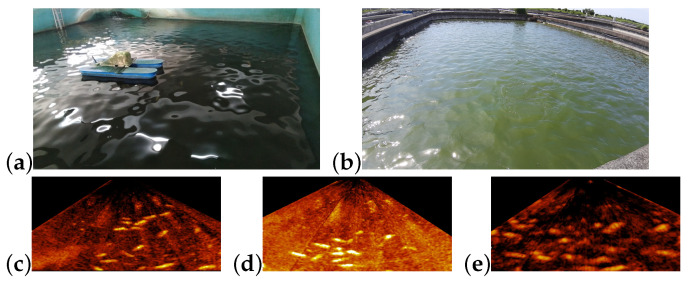
The sonar images of the three test environments, where (**a**,**b**) show the fish farms for test environments E-A,B and E-C, respectively; (**c**–**e**) show examples of the sonar images in test environments E-A, E-B, and E-C, respectively.

The sonar images for this experiment were collected from three environments. Figure 5 shows examples of sonar images collected from the three environments.

- E-A:The first environment is an indoor land-based fish farm with a concrete bottom. The species of the fish in this fish farm is Cyprinus carpio haematopterus.
- E-B: The second environment is the same as the first one except that the gain setting on the sonar system was higher to show more details.
- E-C: The third environment is an outdoor land-based fish farm with a mud bottom. The species of the fish in this fish farm is Pampus argenteus.

**Figure 6:**
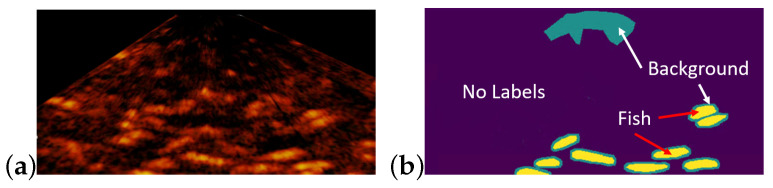
An example of the annotation image: (**a**)sonar image; (**b**) annotation image.

Because it is not easy to precisely identify every fish in a sonar image, only the fish, whose boundary can be unambiguously identified, was annotated. The region around an annotated fish was annotated as the background. A region, where there are sure no fish, was also annotated as the background. Some regions in the annotation image can have no labels. Figure 6 shows an example of the annotation image and Table 1 shows the number of annotated examples.

In this experiment, the backbone network of Mask R-CNN was ResNet101. When Mask R-CNN was trained, the backbone network above the fifth block (included) and the head of Mask R-CNN were fine-tuned by the mini-batch stochastic gradient descent algorithm with at most 200 epochs.

The proposed algorithm was implemented in the Python programming language with software libraries OpenCV, Keras, and TensorFlow. The experiments were performed on a desktop with an Intel Core i7-7700 3.6-GHz CPU, 64-G RAM, and one NVIDIA TITAN RTX GPU card.

### 4.2. Performance Evaluation

In this experiment, the ground truth for a test image does not include ambiguous fish due to annotation difficulty. The average precision (AP) was estimated by five-fold cross-validation. Thus, a high AP indicates that most of the annotated fish are detected and have a high rank in the list of the detected fish. A low AP reveals that many annotated fish are not detected or there are many ambiguous fish, which are not ground-truthed, have a high rank in the list of the detected fish.

#### 4.2.1. Mask R-CNN vs. Mask R-CNN+PreCNN

Table 2 shows the AP${}_{0.5}$ (average precision with the mask IoU 0.5) and the AP${}_{0.75}$ of Mask R-CNN and Mask R-CNN+PreCNN. The threshold for the confidence score in Mask R-CNN was set to 0.2 for calculating the AP. Observations on Table 2 are as follows.

- The AP${}_{0.5}$ of Mask R-CNN is high when the training and the test example are of the same environment.
- The AP${}_{0.5}$ of Mask R-CNN is degenerate when applying Mask R-CNN trained for one test environment to the other two test environments.
- The AP${}_{0.5}$ of Mask R-CNN across environments E-A and E-B is acceptable. Mask R-CNN trained for one test environment can apply to the same environment with a different but proper gain setting on the imaging sonar system.
- The AP${}_{0.5}$ of Mask R-CNN across environments E-A and E-C or across environments E-B and E-C is low. This is because the echoes reflected from the different fish species and the bottom of different materials show different patterns.
- The overall AP${}_{0.5}$ of Mask R-CNN can be improved if the training examples are from the three test environments.
- When the training and test examples are of different environments, Mask R-CNN+ PreCNN is more accurate than Mask R-CNN. Besides, even though Mask R-CNN is fine-tuned on the examples of the three test environments, Mask R-CNN+PreCNN learned on the training example of one single test environment is at least as accurate as Mask R-CNN. That experimental result shows that Mask R-CNN based on the semantic feature map outputted by PreCNN is less dependent on the environment and supports the feasibility of the proposed approach.
- Because the AP${}_{0.75}$ of Mask R-CNN+PreCNN is better, Mask R-CNN+PreCNN can segment fish in a way more consistent with human annotations.

**Figure 7:**
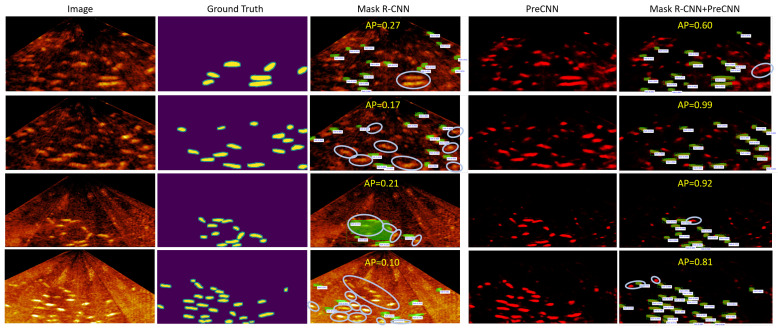
Examples of applying Mask R-CNN and Mask R-CNN+PreCNN across different environments, where a fish in the ground truth and not detected is enclosed by a circle; the threshold of the confidence score for Mask R-CNN is 0.2; the first two rows show applying the model for E-A to E-C and applying the model for E-B to E-C, respectively, and the last two rows show applying the model for E-C to E-A and to E-B, respectively.

As Figure 7 shows, applying Mask R-CNN trained for environment E-A or E-B to environment E-C can miss some fish. A possible cause is that the fish in the sonar image of environment E-C is blurrier and has lower contrast. Applying Mask R-CNN trained for environment E-C to environment E-A or E-B can miss some fish and detect incorrect fish with high confidence scores. In summary, Mask R-CNN+PreCNN is more accurate than Mask R-CNN alone in using for a single test environment and in applying across different test environments.

#### 4.2.2. Mask R-CNN+Image Preprocessing vs. Mask R-CNN+PreCNN

**Figure 8:**
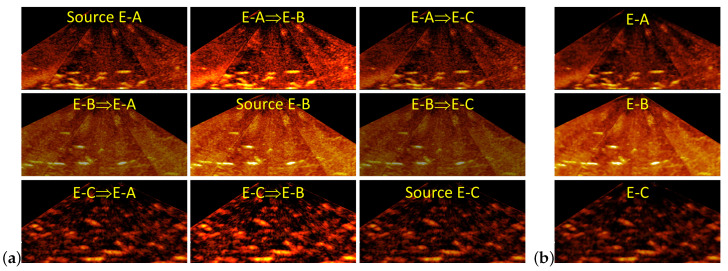
Examples of sonar images processed with contrast stretching and bilateral filtering, where (**a**) shows the source sonar image and the image transformed from the source image into the image for the target environment by contrast stretching; (**b**) shows the source image processed with a bilateral filter.

This experiment compared Mask R-CNN incorporated with contrast stretching and bilateral filtering to Mask R-CNN+PreCNN. The test for Mask R-CNN with contrast stretching evaluates if contrast stretching can transfer the training sonar image collected from one test environment into the training sonar image for the others. Figure 8a shows that the contrast in sonar images can be tuned by contrast stretching. However, by comparing Table 2 and Table 3, Mask R-CNN fine-tuned on the image, which is transformed from the training image for another test environment by contrast stretching, does not improve in crossing different test environments.

The test for Mask R-CNN with bilateral filtering evaluates if Mask R-CNN on the sonar image preprocessed with bilateral filtering is more accurate. Figure 8b shows examples of sonar images processed by a bilateral filter, where the background of the processed sonar image becomes less noisy. However, by comparing Table 2 and Table 3, bilateral filtering does not improve Mask R-CNN in the AP${}_{0.5}$.

Mask R-CNN+PreCNN is more accurate than Mask R-CNN incorporated with contrast stretching and bilateral filtering because PreCNN is a nonlinear mapping from successive sonar-image frames to a semantic feature map.

#### 4.2.3. PreCNN vs. PreCNN with CNN Only

**Figure 9:**
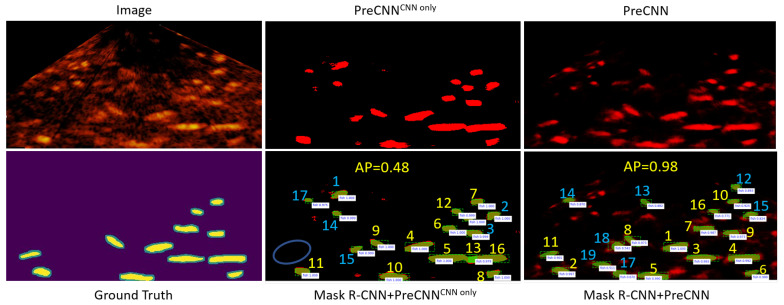
Example results of Mask R-CNN+PreCNN and Mask R-CNN+PreCNN${}^{\mathrm{CNN}\;\mathrm{only}}$, where the number associated with an object is the rank and the object with a blue number is not in the ground truth.

PreCNN only based on the CNN for $\phi_{u}\left(y_{i}X\right)$ without the pairwise potential was also analyzed. This version of PreCNN is referred to as PreCNN${}^{\mathrm{CNN}\;\mathrm{only}}$. According to Table 2 and Table 4, PreCNN${}^{\mathrm{CNN}\;\mathrm{only}}$ is less accurate, especially in Environment E-C. As Figure 9 shows, this is probably because Mask R-CNN+PreCNN${}^{\mathrm{CNN}\;\mathrm{only}}$ often gives high confidence scores to detected fish. Thus, the fish not in the ground truth can have a high rank in the list of detected fish and the AP of the detection result can be lower. The output of PreCNN on fish is smoother. Mask R-CNN+PreCNN can rank the detected fish in a way more consistent with the way of human annotators probably because smooth boundaries are important cues for annotators to identify fish in sonar images.

#### 4.2.4. Experimental Results of YOLOv4

YOLOv4 [51] is a well-known bounding-box object detection model. The training fish for YOLOv4 is only annotated with a bounding box, whereas a training fish for Mask R-CNN and PreCNN requires a mask annotation. The cost of annotating a training fish for YOLOv4 is much lower than that for Mask R-CNN and PreCNN. Table 5 shows that the AP${}_{0.5}$ for YOLOv4 is lower than that for Mask R-CNN and Mask R-CNN+PreCNN. Particularly, the AP${}_{0.5}$ of YOLOv4 sharply deteriorates when YOLOv4 is applied across the test environments E-B and E-C. The mask annotation of training fish is helpful for the detection and segmentation of fish in sonar images.

### 4.3. Segmentation of Overlapping Fish with Mask R-CNN+PreCNN${}^{d}$

**Figure 10:**
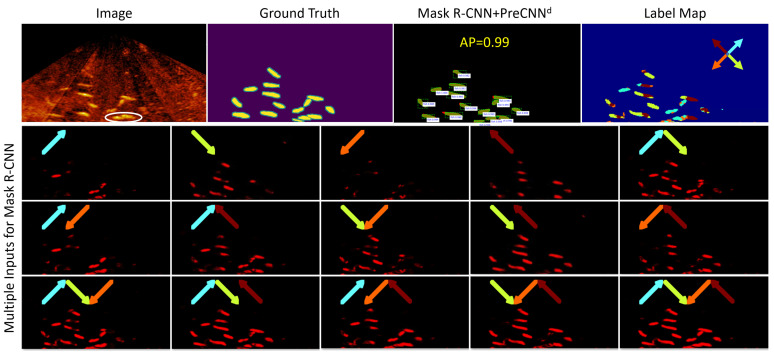
An example of segmenting overlapping fish by Mark R-CNN+PreCNN${}^{d}$, where the overlapping fish are highlighted by a circle and the label map is yielded according to the output Q of PreCNN${}^{d}$.

**Figure 11:**
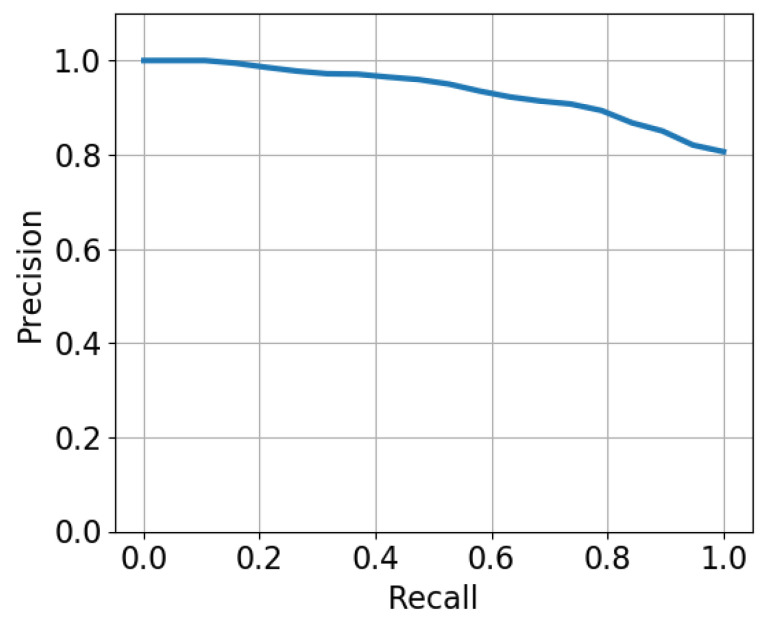
The average precision-recall curve.

In this experiment, sixteen sonar images from environment E-A were selected for testing Mask R-CNN+PreCNN${}^{d}$. Because annotators must definitely identify every fish including overlapping fish, challenging sonar images were not selected. A total of 243 fish including 50 overlapping fish were identified in those images. Figure 10 shows an example of segmenting overlapping fish. Fish locomotion comprises local and global motion and some motion directions are ambiguous within a small receptive field. Thus, it can be seen that there may be multiple labels of motion directions on a fish. Figure 11 shows the average precision-recall curve. All fish can be detected with 20 percent of false positives.

## 5. Conclusions

In this paper, a preprocessing CNN has been proposed to provide “standardized” feature maps for Mask R-CNN for fish segmentation in sonar images. The proposed preprocessing CNN is a semantic segmentation network and integrated with conditional random fields. The preprocessing CNN is aimed at decoupling learning fish instances from learning fish-cultured environments. As a result, the proposed approach can improve Mask R-CNN for segmenting fish in sonar images and can also ease applying Mask R-CNN across fish-cultured environments. In the future, the efficiency of the proposed framework will be improved by developing a lightweight fish-instance segmentation network on the proposed preprocessing CNN.

## Figures and Tables

**Table 1:** The specifications of the dataset.

Test Environment	E-A	E-B	E-C
Total number of sonar images	50	60	35
Total number of annotated fish	522	794	360

**Table 2:** The AP${}_{0.5}$ and AP${}_{0.75}$ of Mask R-CNN and Mask R-CNN+PreCNN.

	Test (AP${}_{0.5}$)					
	Mask R-CNN			Mask R-CNN+PreCNN		
Training	E-A	E-B	E-C	E-A	E-B	E-C
**E-A**	0.86 ± 0.06	0.71 ± 0.07	0.37 ± 0.07	0.97 ± 0.01	0.96 ± 0.03	0.75 ± 0.07
**E-B**	0.66 ± 0.11	0.84 ± 0.06	0.17 ± 0.07	0.92 ± 0.04	0.99 ± 0.01	0.80 ± 0.02
**E-C**	0.41 ± 0.04	0.17 ± 0.05	0.96 ± 0.01	0.85 ± 0.05	0.91 ± 0.03	0.95 ± 0.02
**E-AB**	0.84 ± 0.04	0.84 ± 0.06	0.45 ± 0.08			
**E-BC**	0.71 ± 0.09	0.83 ± 0.06	0.86 ± 0.06			
**E-AC**	0.80 ± 0.06	0.61 ± 0.13	0.83 ± 0.04			
**E-ABC**	0.81 ± 0.08	0.79 ± 0.04	0.76 ± 0.03			
	**Test (AP${}_{0.75}$)**					
	**Mask R-CNN**			**Mask R-CNN+PreCNN**		
**Training**	**E-A**	**E-B**	**E-C**	**E-A**	**E-B**	**E-C**
**E-A**	0.47 ± 0.14	0.13 ± 0.03	0.04 ± 0.03	0.88 ± 0.05	0.84 ± 0.04	0.57 ± 0.10
**E-B**	0.11 ± 0.07	0.47 ± 0.04	0.01 ± 0.01	0.75 ± 0.07	0.89 ± 0.05	0.60 ± 0.08
**E-C**	0.10 ± 0.03	0.01 ± 0.01	0.71 ± 0.12	0.53 ± 0.06	0.70 ± 0.07	0.84 ± 0.03

**Table 3:** The AP${}_{0.5}$ of Mask R-CNN with image preprocessing.

	Test (AP${}_{0.5}$)					
	Mask R-CNN+Contrast Stretching			Mask R-CNN+Bilateral Filtering		
Training	E-A	E-B	E-C	E-A	E-B	E-C
**E-A**	0.86 ± 0.06	0.67 ± 0.04	0.36 ± 0.05	0.86 ± 0.03	0.68 ± 0.04	0.38 ± 0.04
**E-B**	0.61 ± 0.04	0.84 ± 0.06	0.15 ± 0.06	0.55 ± 0.13	0.84 ± 0.03	0.09 ± 0.08
**E-C**	0.40 ± 0.06	0.08 ± 0.04	0.96 ± 0.01	0.41 ± 0.02	0.11 ± 0.03	0.89 ± 0.06

**Table 4:** Experimental results of Mask R-CNN+PreCNN${}^{\mathrm{CNN}\;\mathrm{only}}$.

	Test (AP${}_{0.5}$)		
	Mask R-CNN+PreCNN${}^{\mathrm{CNN}\;\mathrm{only}}$		
Training	E-A	E-B	E-C
**E-A**	0.90 ± 0.04	0.73 ± 0.06	0.62 ± 0.06
**E-B**	0.85 ± 0.02	0.79 ± 0.03	0.60 ± 0.05
**E-C**	0.79 ± 0.02	0.71 ± 0.07	0.57 ± 0.03

**Table 5:** Experimental results of YOLOv4.

	Test (AP${}_{0.5}$)		
Training	E-A	E-B	E-C
**E-A**	0.57 ± 0.04	0.56 ± 0.01	0.45 ± 0.02
**E-B**	0.47 ± 0.06	0.59 ± 0.04	0.28 ± 0.04
**E-C**	0.40 ± 0.02	0.25 ± 0.02	0.47 ± 0.02

## Data Availability

The study did not report any data.
